# Auriculotherapy in prevention of migraine attacks: an open randomized trial

**DOI:** 10.3389/fneur.2023.1193752

**Published:** 2023-05-22

**Authors:** Mireille Michel-Cherqui, Sabrina Ma, Marguerite d’Ussel, David Ebbo, Antoinette Spassova, Carine Chaix-Couturier, Barbara Szekely, Marc Fischler, Nicolas Lemaire, Morgan Le Guen

**Affiliations:** ^1^Department of Anesthesiology, Hospital Foch, Suresnes, France; ^2^Department of Medicine, Université Versailles Saint-Quentin en Yvelines, Versailles, France; ^3^Department of Pain Medicine, Hospital Saint Joseph, Paris, France; ^4^General Practice, Maison-Alfort, France; ^5^General Practice, Montigny le Bretonneux, France; ^6^Axonal-Biostatem, Nanterre, France

**Keywords:** headache, auriculotherapy, migraine, randomized controlled trial, pain measurement, life quality

## Abstract

Use of auriculotherapy to prevent episodic migraine pain has seldom been reported. The aim of this open study was to show that three sessions of auriculotherapy, 1 month apart, using semi-permanent needles decrease frequency and intensity of an attack in patients presenting episodic migraine. A total of 90 patients were randomized to the treatment group (AUR group, *n* = 58) or the control group (C group, *n* = 32). Four patients dropped out during the study (three in the AUR group and one in the C group). The number of days with migraine and non-migraine headache was similar when the analysis focused on the 3 months of the study or on the difference in each group of this number between the 3 months preceding the inclusion and the 3 months of the study (*p* = 0.123). AUR group patients had fewer days with non-migraine headache (*p* = 0.011) and took less Triptans (*p* = 0.045) than group C. Number of days with migraine, sum of the pain intensities of all migraines and non-migraine headaches, and total number of analgesics taken, other than triptan, were similar between groups. MIDAS score decreased with time in the AUR group while it increased in the C group whether in absolute values (*p* = 0.035) or as categories (*p* = 0.037). These contrasted results should lead to further study of the effectiveness of auriculotherapy for the prevention of migraine.

**Clinical trail registration**: Protocol registered on the Clinicaltrials.gov, website (January 30, 2017, NCT03036761).

## 1. Introduction

Migraine is a common disabling primary headache disorder and a frequent pain complaint in general practice and Pain centers. Patients with migraine also describe frequent non migraine headaches.

Migraine is also associated with significant disability, reduced health-related quality of life, overuse of acute pain medications (1) and is responsible for a high economic burden (2).

Preventive treatment of migraine is often difficult, with significant numbers of patients not responding to pharmacologic management. Randomized controlled trials showing a significant effect in the treatment specifically of chronic migraine have been published at present only for topiramate and onabotulinumtoxin A (3, 4). Nonpharmacologic treatment can be several tools, like mind body techniques, biofeedback, physical therapy, acupuncture. Among them, auriculotherapy which has a helpful place in chronic pain (5), could play a role. Auriculotherapy, developed by the French Doctor Paul Nogier in 1957, is a treatment method based on normalizing body dysfunction by treating specific points on the external ear. Auriculotherapy exercises probably activate neuromodulation on the central neural system via the innervation of the auricle which comes from trigeminal, vagal, and spinal nerves (6–9).

Randomized controlled trials have already demonstrated that the puncture of a specific point, with a semi-permanent needle, induces a significant decrease in the intensity of pain during migraine attacks (10, 11).

Two studies have reported that auriculotherapy decreases the occurrence of headaches and obtains significant positive results on frequency, intensity, and duration of migraines (12, 13). However, further studies are necessary to clarify some issues, session spacing and evaluation of the quality of life and of the use of specific drugs like triptans.

The aim of our study is to show that three sessions of auriculotherapy, 1 month apart, using semi-permanent needles can decrease the frequency and intensity of attacks in patients presenting episodic migraine. For our primary objective, we tested the hypothesis that our protocol reduces the number of days with migraine and non-migraine headaches compared to controls in patients presenting with episodic migraines. For our secondary objectives we tested the hypothesis that it decreases medication intake and improves the quality of life of patients presenting with episodic migraine.

## 2. Materials and methods

### 2.1. Trial design

This randomized, controlled multicenter open study was performed in two tertiary care university hospitals and in two private offices in accordance with the Declaration of Helsinki. Ethical approval for this study was provided by the Ethical Committee Kremlin Bicetre (N° 16–025; September 27, 2016; Chairperson AM TABURET, MD), Paris, France. The study was designed in accordance with the Consolidated Standards of Reporting Trials (CONSORT 2010) and published on the Clinical.trials.gov website (January 30, 2017; NCT03036761). The procedure of Auriculotherapy was conducted according to STRICTA guidelines (14).

### 2.2. Participants

We studied female patients 18 to 80-years-old, with a diagnosis of episodic migraine (15) and presenting at the consultation with a filled out 3-month headache diary since the three participating centers asked patients with migraine to fill out a 3-month diary before attending the first consultation.

Patients were eligible if their symptoms had been present for more than 6 months, if they were taking a stable acute medication, and if no migraine-prophylactic medication or stable migraine-prophylactic medication had been taken for more than a month.

Patients were not included in the following cases: other primary or secondary headaches, severe neurologic or psychiatric disorders including opioid- or tranquilizer-dependency, current participation in another clinical trial, planned change in treatment that could interfere with the study, inability to keep a headache diary, pregnancy or breast-feeding at the time of the inclusion. Patients who had less than six or more than 45 headache days during the 3-month baseline period were also not included. Lastly, patients were not included if they had ear infection or abnormality, valvular prosthesis, hemophilia or anticoagulation treatment and auricular treatment for this indication in the year before the inclusion.

During the first consultation patients were enrolled after verbal explanation of the protocol plus delivery of a written information sheet. They signed an informed consent form after a suitable interval. Enrollment was the sole responsibility of the investigating team.

### 2.3. Investigators

Auriculotherapy was performed by six experienced physicians, all of them graduated from the “Diplome Inter-Universitaire d’Auriculotherapie-Neuromodulation Auriculaire” of Paris-Saclay University. All had more than 7 years of auriculotherapy practice.

### 2.4. Procedure

Patients were carefully informed about the protocol and signed an informed consent form. Then, they were informed if they were recruited in the treated group (AUR group) or in the control group (C group) who received no treatment in relation to the study.

AUR group patients benefited from three auriculotherapy sessions at one-month intervals. Each auriculotherapy session consisted in implanting at the level of each pavilion of the ear semi-permanent sterile single-use needles in nickel-free stainless steel with a diameter of 0.7 mm and a length of 2 mm (ASP® needles classic, Sedatelec SA, 69540 Irigny, France). Prior to the procedure, the researcher performed careful hand disinfection and allowed the patient to be relaxed and prone. The insertion was done bilaterally after application of the antiseptic (alcohol at 70°), then drying in the open air for at least 2 min.

**Figure 1:**
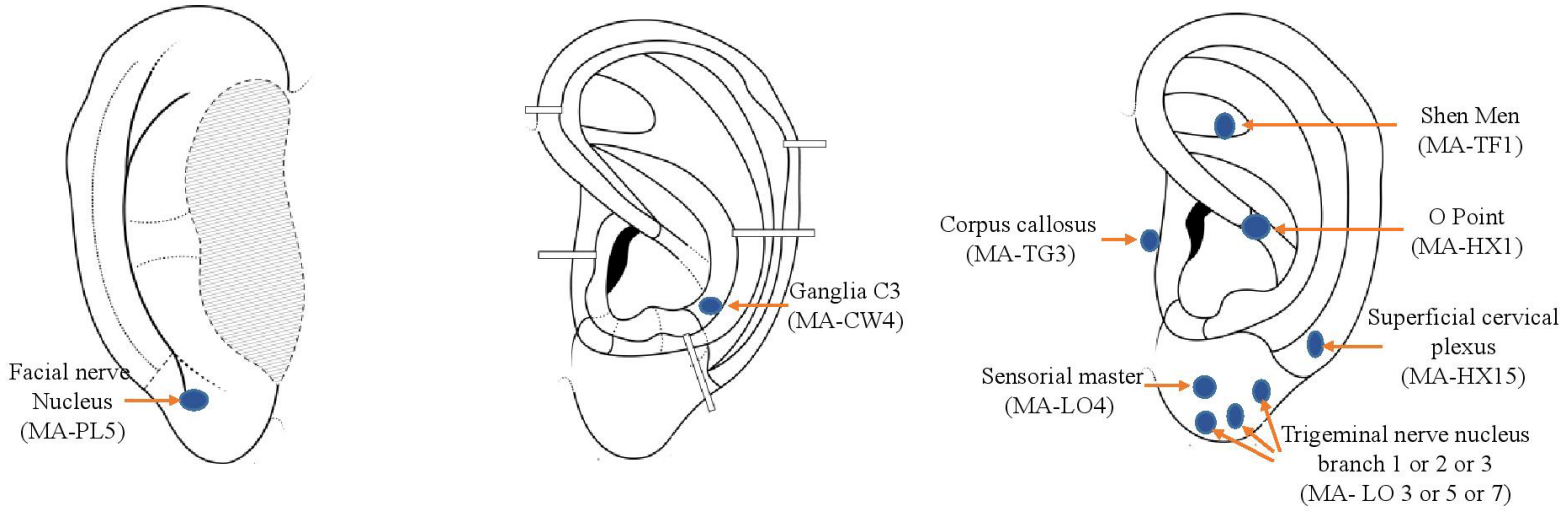
Treated points. Position of treated points (https://aurimatrix.com/fr; assessed on March 1, 2023) Each point is named according to the French classification points taught by Nogier and Alimi (16) and in brackets according to the Auricular Acupuncture Nomenclature of the World Health Organization (17).

Figure 1 presents treated points {https://aurimatrix.com/fr; assessed on March 1st, 2023}. Each point is named according to the French classification points taught by Nogier and Alimi (16) and in brackets according to the Auricular Acupuncture Nomenclature of the World Health Organization (17). In case of unilateral migraines, the ipsilateral ear was treated with seven points (sensorial master, Trigeminal nerve branch 1 or 2 or 3 according to the predominant location of the migraine attacks, ganglia C3, Facial nerve, shen men, corpus callosus) and four points were pricked on the contralateral ear (Shen men, Sensorial master, Corpus callosus and O). In case of bilateral migraines, the same seven-point protocol was used on both ears. Physicians had the possibility to treat an extra point depending on the patient’s symptomatology (i.e., anxiety, catamenial migraines).

The accuracy of the location of the points was confirmed by their tenderness on palpation, and/or their decreased resistance electrical power, tested with a specific measuring device, the Pointoselect digital acupuncture point detector (http://www.schwa-medico-france.fr/). The use of the acupuncture point detector was left to the discretion of the auriculotherapist.

Participants were asked not to apply any pressure on the needles or manipulate them. They were informed that the needles would fall off by themselves. If any adverse reaction such as local redness or increased pain occurred, the participants were instructed to contact the study center.

Final evaluation was performed 4 months after randomization (i.e., 1 month after the last auriculotherapy treatment).

### 2.5. Measurement and data collection

**Figure 2:**
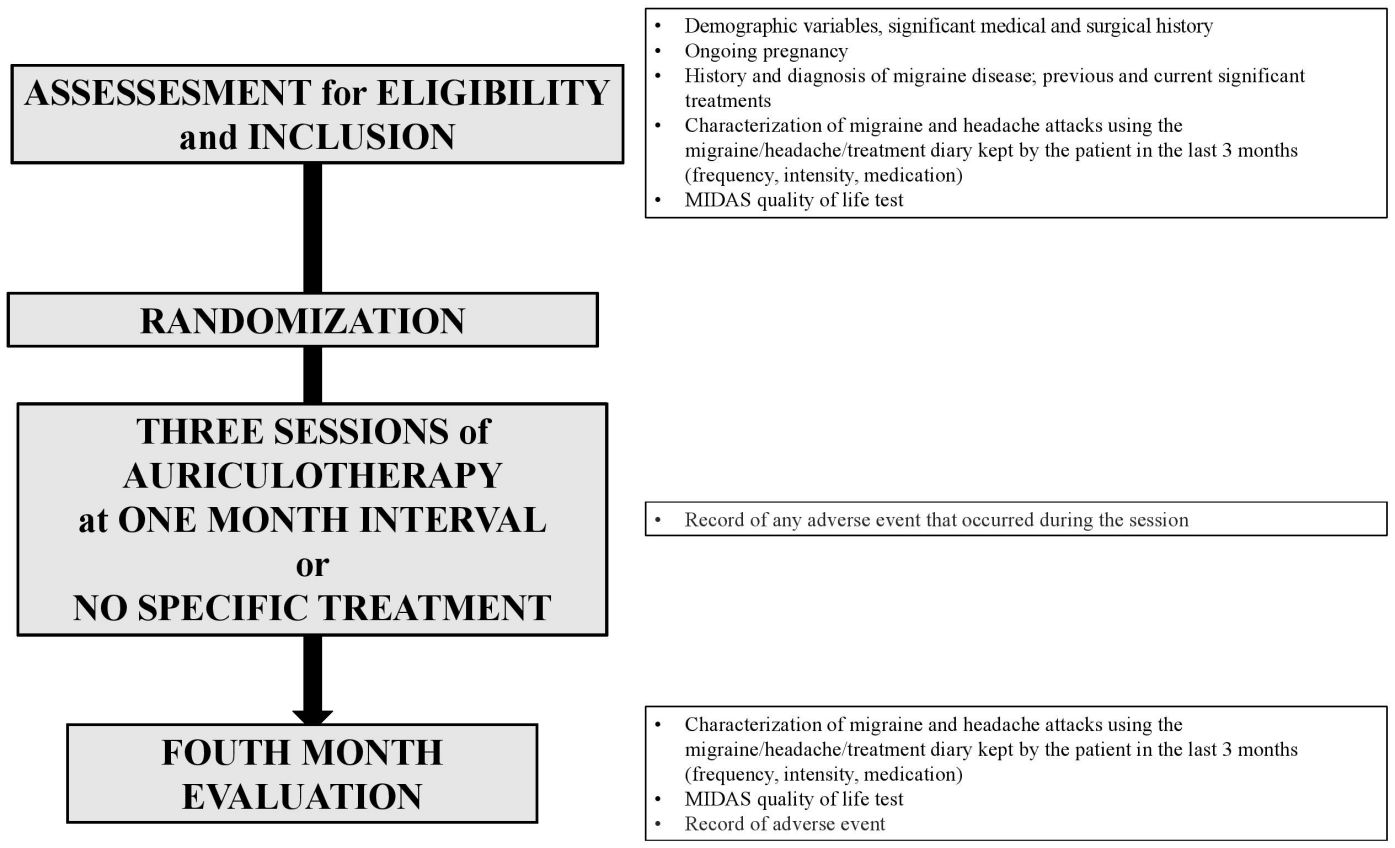
Data collection.

Figure 2 summarizes data collection.

Migraine days, non-migraine headache days, use of triptans and other pain medication, severity of headache, and quality of life were obtained via 3-month diaries: a pre-inclusion diary filled out by the patients during the 3 months prior to the inclusion to the study, a post-inclusion diary filled out during the study.

A day was considered a day with headache when the pain episode lasted more than 4 h, or if the pain episode was suppressed by a specific treatment known to the patient. Many patients differentiate migraine and cephalalgia headaches, but a special item “undifferentiated headache” could be used in case of doubt. The intensity of each pain episode was evaluated by the patient on a numeric scale of 1 to 3 (1 = low, 2 = moderate, 3 = intense). The cumulative intensity of pain was evaluated by the calculation of the sum of the pain intensities of all the pain episodes. Quality of life was assessed via the migraine disability assessment test (MIDAS) (18).

Adverse events, notable pain using a 11-point scale for patient self-reporting of pain (from 0 = pain to 10 = most severe pain) and tolerance to the needle placement was retrieved at the end of each session, tolerance to the needles during the month following the treatment was retrieved during the following session, by the physician in charge of the consultation.

### 2.6. Outcomes

The main outcome was the number of days with migraines and non-migraine headaches during the 3 months of auriculotherapy treatment. Non-migraine headaches could be reported in the diary as cephalalgia or as undifferentiated headache. Secondary outcomes of the study were the number of days with migraine, the number of days with non-migraine headache, the cumulative dose of analgesics, the cumulative intensity of painful episodes (migraines and non-migraine headaches) and the quality of life during the 3 months of auriculotherapy treatment. Pain induced by auriculotherapy treatment was assessed using a numeric pain score from 0 (no pain) to 10 (maximal pain). The pain was evaluated during the needle placement and a mean pain was evaluated during the month following the treatment.

### 2.7. Sample size

As auriculotherapy had previously been used by the investigators routinely in this indication, the sample size was calculated based on our experience. We assumed that the number of migraine and non-migraine headache days would be on average 8 per 3 months in the control group and that the standard deviation in this population would be around 3. Assuming that the number of crisis days would fall by half in the treated group with a standard deviation of 2.5. Based on this assumption, with a risk α of 0.05 and a power 1 − β of 0.80, the numbers being unbalanced (⅔ vs. ⅓), considering the risk of attrition of 20%, we obtained a sample size of 60 patients randomized in the treated group (AUR group) and 30 in the control group (C group).

### 2.8. Randomization and implementation

The random allocation sequence, with a 1:3 ratio and blocks of 10, was generated through the web site “Randomization.org,” of which the generator can be considered to be validated by experience. Scratch cards were generated and printed on a special printer; they were then kept in a secure location until used. Concealed scratch cards were retrieved from the secure location by a member of the research team and brought to the physician as soon as the patient’s consent for the study had been obtained.

### 2.9. Statistical methods

Number (percentage) is reported for categorical variables. Mean ± standard deviation or median [25^th^-75^th^ quartiles] are reported for quantitative variables according to the normal or not-normal distribution assessed using the Shapiro–Wilk test.

Three populations were defined: (1) the Intent-to-Treat (ITT) population which corresponded to all included and randomized patients in the study, their main characteristics at inclusion were analyzed in the group assigned to them at randomization; (2) the Per-protocol (PP) population which corresponded to all patients in the ITT population without major protocol deviations, primary and secondary outcomes were analyzed in this population; (3) the Tolerance population (T) which corresponded to all patients who had at least one auriculotherapy session. Results are reported for ITT and T populations.

Comparison between the 2 groups was performed using: (1) the student t-test for normally distributed quantitative variables or the non-parametric Wilcoxon test in the opposite case; (2) the Chi2 test for qualitative variables or Fisher’s exact test. A mixed model analysis of covariance (ANCOVA) adjusted to the inclusion value was also used to analyze the efficacy outcomes; the adjusted mean of the differences between the groups was calculated. Finally, for the main criterion, the following relevant variables at inclusion were selected in a multivariate model with the group and the main inclusion values: age, length of time with migraine, preventive treatment, menopause and triptan use.

The significance level is set at 5% for all tests. Statistical analyses were performed using SAS® software, version 9.4 (SAS Institute, NC, Cary, United States).

The datasets generated and analyzed during the current study are available upon reasonable request.

## 3. Results

The study was conducted between January 18, 2017, and March 22, 2021.

**Figure 3:**
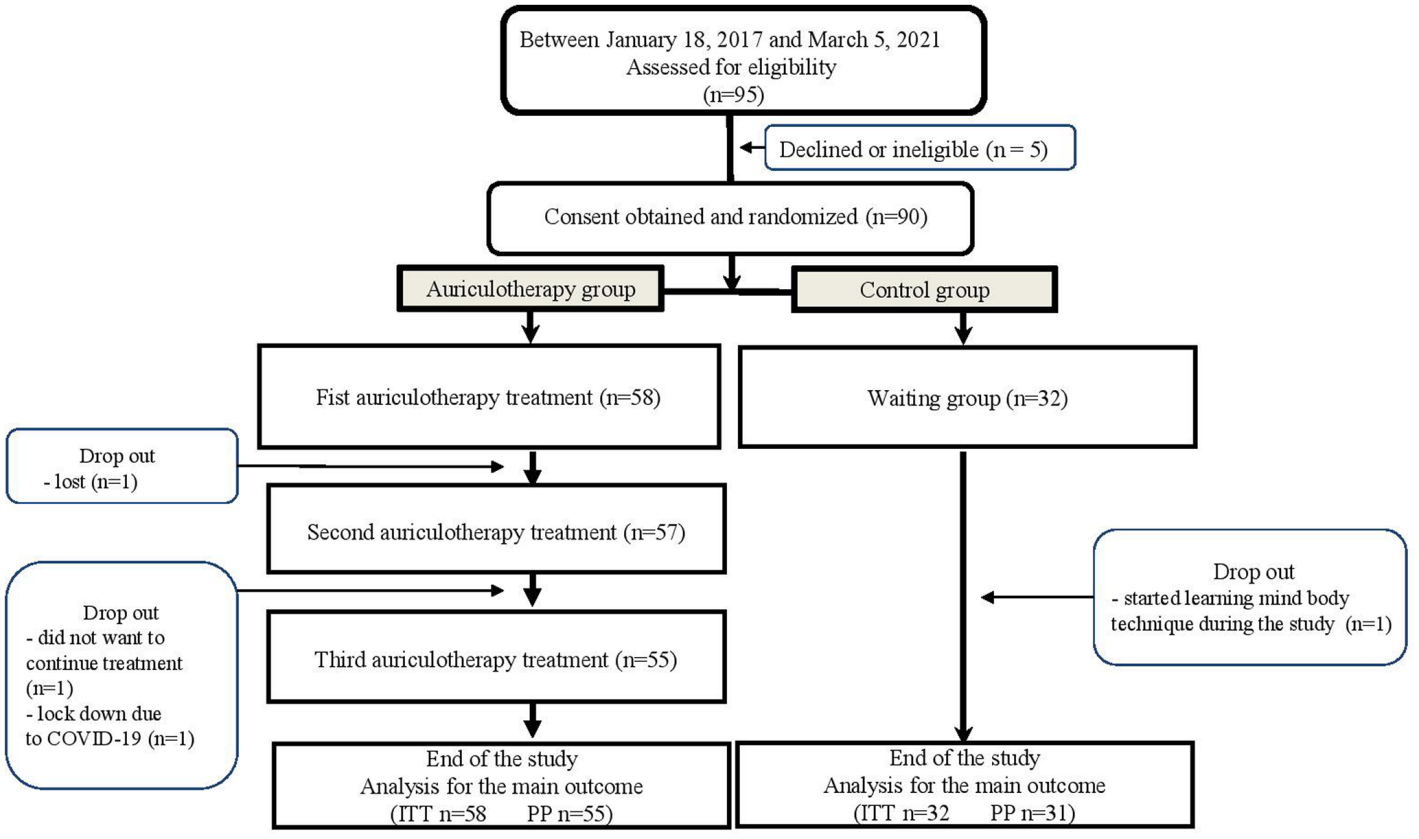
Flow chart ITT: Intent-to-Treat population PP: Per-protocol population.

A total of 90 patients were randomized to the AUR group (*n* = 58) or the C group (*n* = 32). Four patients dropped out during the study (three in the AUR group and one in the C group) (Figure 3).

Repartition of control and treated patients was similar among centers (*p* = 0.266). Duration of participation in the study was similar between groups (3.3 [3.0; 3.4] months in the AUR group and 3.0 [3.0; 3.3] in the C group; *p* = 0.070).

In the AUC group 93.9% of the patients wanted to pursue the auricular treatment on a regular basis at the end of the study.

### 3.1. Participants

There were no significant differences between groups regarding demographic, morphometric, and headache characteristics (Table 1).

**Table 1:** Baseline characteristics of the patients (Intention to treat population).

	AUR group *n* = 58	C group *n* = 32	*p*
Age, years	44.2 ± 11.8	44.4 ± 11.5	0.944
Hypertension	7 (12.1%)	1 (3.1%)	0.251
Diabetes	1 (1.7%)	0 (0.0%)	1
Hormonal status			
Menopause	20 (34.5%)	11 (34.4%)	0.992
Oral contraception			
Combined estrogen/progestogen	6 (10.3%)	2 (6.3%)	0.707
Progestogen contraception	8 (14.0%)	3 (9.4%)	0.740
Migraine			
Length of time with migraine, years	21 [15; 30] {1}	25 [16; 32]	0.535
Aura	20 (34.5%)	5 (15.6%)	0.056
Preventive treatment	19 (32.8%)	10 (31.3%)	0.883
Acute treatment			
At least one drug	58 (100%)	32 (100%)	NA
Triptan			
Use	44 (75.9%)	24 (75.0%)	0.927
Intake in the last 3 months, number	9 [0; 17]	11 [0; 21]	0.814
Frequency and intensity of migraine and headache during the last 3 months			
Migraine, number of days	15 [10; 24]	17 [11; 29]	0.505
Non migraine headache, number of days	0 [0; 6]	1 [0; 6] {1}	0.456
Undifferentiated pain, number of days	0 [0; 0] {3}	0 [0; 0]	0.794
Total of days with pain	21 [13; 30]	24 [15; 30]	0.489
Sum of pain intensities	37 [26; 58] {2}	33 [26; 66]	0.921
MIDAS (Migraine Disability Assessment Test)			
Quantitative responses			
Question 1*	0 [0; 3] {7}	0 [0; 2] {0}	0.475
Question 2**	5 [0; 12] {8}	5 [0; 9] {0}	0.694
Question 3***	2 [0; 7] {3}	2 [0; 6] {1}	0.800
Question 4****	6 [2; 12] {5}	6 [1; 12] {2}	0.856
Question 5*****	6 [1; 12] {5]	2 [0; 9] {1}	0.107
Total (Questions 1–5)	20 [11; 39] {3}	21 [6; 33] {1}	0.390
Classes	{3}	{1}	0.121
I (0–5)	7 (12.7%)	6 (19.4%)	
II (6–10)	5 (9.1%)	5 (16.1%)	
III (11–20)	17 (30.9%)	3 (9.7%)	
IV (≥ 21)	26 (47.3%)	17 (54.8%)	

Results are presented as number (percentage) for categorical variables and mean ± standard deviation or median [25th–75th quartiles] for quantitative variables.

Number of missing values are presented as {}.

* Question 1. On how many days in the last 3 months did you miss work or school because of your headaches?

** Question 2. How many days in the last 3 months was your productivity at work or school reduced by half or more because of your headaches? (Days counted in question 1 are not included).

*** Question 3. On how many days in the last 3 months did you not do household work (such as housework, home repairs and maintenance, shopping, caring for children and relatives) because of your headaches?

**** Question 4. How many days in the last 3 months was your productivity in household work reduced by half or more because of your headaches? (Days counted in question 3 are not included).

***** Question 5. On how many days in the last 3 months did you miss family, social or leisure activities because of your headaches?

### 3.2. Auriculotherapy sessions

Treated patients received auriculotherapy treatment according to the protocol, and especially according to the unilateral or bilateral character of the migraines. Detection of points using a pointo select was achieved in 19.9% of the sessions. An additional point was treated in eight cases: the liver-biliary tract point (MA-SC7) in four cases; the stomach point (MA–SC8) in two cases in patients presenting with severe nausea during migraine attacks; the ACTH (MA-IT2) point once and the FSH-LH (MA-LO2) point once.

### 3.3. Outcomes

ITT-analysis indicated no significant difference in the number of days with migraine and non-migraine headache (Table 2) when the analysis focused on the 3 months of the study or in the difference in each group of this number between the 3 months preceding the inclusion and the 3 months of the study (*p* = 0.123). Main variables at inclusion (age, length of time with migraine, preventive treatment, menopause and triptan use) did not interfere with the main outcome (*p* > 0.10).

**Table 2:** Comparison of main and secondary outcomes between auriculotherapy and control groups (Intention to treat population).

	AUR group *n* = 58	C group *n* = 32	Difference between groups	*p*
Main outcome				
Number of days with migraine and non-migraine headache				
Absolute number*	18 [12;27]{3}	21 [13;29]{1}	−3.2 [−7.4; 0.9]	0.123
Variation**	−3 [−9; 2]{3}	1 [−6; 5]{1}		
Secondary outcomes				
Number of days with migraine				0.738
Absolute number*	13 [7; 22]{4}	13 [11; 26]{2}	0.6 [−3; 4.2]	
Variation**	−2 [−6; 1]{4}	−3 [−8; 4] {2}		
Number of days with non-migraine headache				**0.011**
Absolute number*	0 [0; 4] {5}	1 [0; 12]{2}	−4.0 [−7.1; −0.9]	
Variation**	0 [0; 0] {5}	0 [−2; 6]{3}		
Sum of the pain intensities of all migraines and non-migraine headaches				0.297
Absolute number*	32 [22; 45]{3}	31 [24; 62]{1}	−4.0 [−11.5; 3.6]	
Variation**	−5 [−15; 5]{4}	0 [−9; 8]{1}		
Triptan use, total number of triptan taken				**0.045**
Absolute number*	8 [0; 16]{2}	10 [0; 22]{1}	−3.4 [−6.7; −0.1]	
Variation**	0 [−5; 0]{2}	0 [−1; 3]{1}		
Another antalgic use, total number of another antalgic taken				0.921
Absolute number*	11 [4; 17]{2}	13 [3; 27]{1}	−0.5 [−10.1; 9.1]	
Variation**	−2 [−9; 2] {2}	0 [−8; 6]{1}		

Bold values correspond to *p* >0.05.

Results are presented as median [25th-75th quartiles].

Number of missing values are presented as {}.

Absolute number**: Absolute value reported during the 3 months of the study.

Variation**: Variation between the 3 months before inclusion and during the 3 months of the study.

Similar analysis showed that patients having been treated by auriculotherapy had fewer days with non-migraine headache (*p* = 0.011) and took less triptan pills (*p* = 0.045) than control patients. Number of days with migraine, sum of the pain intensities of all migraines and non-migraine headaches, and total number of analgesics taken, other than triptan, were similar between groups (Table 2). MIDAS score decreased with time in the AUR group while it increased in the C group whether in absolute values (*p* = 0.035) or as categories (*p* = 0.037) (Table 3).

**Table 3:** MIDAS scores (Intention to treat population).

	AUR group *n* = 58	C group *n* = 32	Difference between groups	*p*
MIDAS, score				
At the end of the 3 months of the study				
Question 1	0 [0; 1]{10}	0 [0; 1]{4}	−1.2 [−2.4; −0.1]	**0.035**
Question 2	2 [0; 6]{11}	4 [1; 11]{4}	−4.4 [−8.3; −0.5	**0.023**
Question 3	2 [0; 5]{10}	2 [1; 5]{4}	−1.3 [−3.4; 0.9]	0.242
Question 4	1 [0; 10]{11}	3 [0; 6]{4}	0.4 [−2.3; 3.1]	0.787
Question 5	1 [0; 7]{11}	2 [0; 7]{4}	−2.3 [−5.4; 0.8]	0.145
Total	8 [2; 24]{6}	15 [9; 22]{3}	−9.3 [−17.9;-0.6]	**0.035**
Variation between the 3 months before inclusion and during the 3 months of the study	−8 [−19; −1]{7}	3 [−17; 11] {3}		
MIDAS, classes	{8}	{4}		0.202
At the end of the 3 months of the study				
I	19 (38.0%)	5 (17.9%)		
II	5 (10.0%)	5 (17.9%)		
III	8 (16.0%)	8 (28.6%)		
IV	18 (36.0%)	10 (35.7%)		
Variation between the 3 months before inclusion and during the 3 months of the study	{1}	{1}		**0.037**
Amelioration	19 (38.8%)	6 (21.4%)		
Stabilization	28 (57.1%)	16 (57.1%)		
Regression	2 (4.1%)	6 (21.4%)		

Bold values correspond to *p* >0.05.

Results are presented as median [25th–75th quartiles].

Number of missing values are presented as {}.

Absolute number**: Absolute value reported during the 3 months of the study.

Variation**: Variation between the 3 months before inclusion and during the 3 months of the study.

### 3.4. Safety and tolerability

Self-reported pain during needle placement and during the month following treatment is reported in Table 4. Other minor adverse events included local redness (21 cases), local pruritus (7 cases), rapidly resolutive local infection (3 cases), local hematomas (2 cases), light dizziness or fatigue during the first days following the treatment (3 cases), and one very intense migraine attack 24 h after the treatment.

**Table 4:** Pain related to placement of the needles for each of the three treatments (Tolerance population).

	Treatment 1 (*n* = 58)	Treatment 2 (*n* = 57)	Treatment 3 (*n* = 53)
Acute pain during needle placement, numeric pain score	2.6 ± 2.0	3.4 ± 2.4 {1}	2.8 ± 2.4 {5}
Mean Pain due to the needles during the month following treatment. Numeric pain score	1.9 ± 2.3 {1}	1.8 ± 2.3 {3}	1.7 ± 2.3 {5}

Results are presented as mean ± standard deviation.

Number of missing values are presented as {}.

Numeric pain score: score from 0 (no pain) to 10 (maximal pain).

## 4. Discussion

Auriculotherapy, practiced as three sessions 1 month apart, using semi-permanent needles, does not significantly decrease the number of headache days. However, auriculotherapy significantly decreases triptan use and improves MIDAS score. This technique carries no severe major event and interestingly almost all patients wanted to pursue the auricular treatment on a regular basis at the end of the study.

### 4.1. Background on auriculotherapy and its mechanisms of efficacy

Acupuncture has been shown to exert its effects through a modulation of local, locoregional and global neurobiological mechanisms. Although the exact mechanisms of auriculotherapy are far from being fully understood, the triple innervation of the auricle provides a hypothesis for a locoregional and global modulation of the neurobiological mechanisms involved in migraines and for some of them close to the acupuncture mechanism. Modulation of trigeminal nuclei via the auricular branch of the trigeminal nerve could regulate neuroinflammation and neuronal sensitization found in migraine patients. This mechanism has been proposed in acupuncture studies for the scalp acupoint (19). The role of vagal stimulation is more specific to auriculotherapy. Stimulation of the concha, via the auricular branch of the vagus nerve and the activation of the nucleus tractus solitari, induces an effect on visceral organs and on brain structures. Neuroimaging studies have shown that this stimulation is responsible for an activation of the locus ceruleus, the insula, the thalamus and inhibition of limbic structures (20, 21). These vagal afferents modulate different neurotransmitter systems and possibly play a role in pain and inflammation regulation. More recently it has been proposed that various clinical manifestations of migraine could be the result of abnormal brain network connections (19). Vagal stimulation could improve resting state functional connectivity (22).

### 4.2. Relation to previous studies

Although auriculotherapy has been described since the fifties, there are few studies on migraine and many of them concern the efficacy of the technique during migraine attacks (10, 11). To our knowledge, only two studies have focused on the prevention of headaches using auriculotherapy and obtained significant positive results (12, 13). Ceccherelli et al. compared somatic and ear acupuncture for treatment of migraine and reported that pain at the end of 8 weeks of therapy was significantly reduced (12) while Habibabadi et al. compared auricular acupuncture with semi-permanent (ASP) needles and routine treatments with a decrease in the level of pain and the frequency of migraine headaches from the second week after the intervention (13).

Due to the lack of some details, it is difficult to specify the severity of patients treated by Ceccherelli et al. while the patients in Habibabadi’s study are better described with about three to four migraine days a week, aged about 37 years old and having suffered from migraines for about 10 years. These characteristics can be compared with those of our patients: about 16 migraines per 3 months, aged about 44 and a history of 23 years with migraine.

Comparison between these studies and our study is made difficult by differences in methodology: frequency of treatment, use of an electric detector and choice of the treated points especially.

Spacing a treatment to once a month comes from our experience and is more comfortable for the patients, but we could hypothesize whether increasing the frequency of treatment could have improved our results.

The use of a detector is a subject of discussion. Body acupuncture research on acupuncture points (or acupoints) have revealed that pathological body conditions cause considerable changes in skin conductance or impedance at acupoints. Skin electrical resistance depends on the activity of the sympathetic nervous system and is also a result of the release of the neuropeptides substance P and calcitonin gene-related peptide during neurogenic inflammation in a referred pain area (23), a mechanism which could also be at play in auriculotherapy. Detection of points of decreased resistance is frequent in auriculotherapy studies and investigation of these points and their relation to their tenderness and an underlying pathology has been published in international literature (24–26). Oleson reported that Tender points and area of increased skin conductivity correspond with medical diagnosis in 75% of the cases (27). Even in the absence of definitive arguments, using a device systematically to determine the points of less resistance could perhaps also have improved our results.

Regarding the choice of the points, Nogier identified auricular points empirically and described a “somatotopic” organization of the body represented on the human pavilion of the ear and published the first map of the ear in 1957 (28). Since Nogier published his first chart, there have been many maps describing the position of the auriculotherapy points, and despite the willingness of many scientific organizations to propose a common map and a set of common points, many differences persist between schools and countries (World Health Organization (WHO). WHO Report of the Working Group on Auricular Acupuncture Nomenclature. Geneva: WHO; 1990). We decided, in this clinical trial, to choose an association of systematic auricular points. The choice of these points was based on the French cartography taught by Nogier and responded to a basic physio pathological vision of the migraine: activation of the trigemino-vascular system, the repetition of attacks that characterizes migraine disease results from a defect in cerebral excitability that is of genetic origin and that makes the migraine sufferer more vulnerable to multiple triggering factors that are characterized by a change of state (fatigue, stress, lack of sleep, muscular tension). This physiopathology also includes the implication of the limbic system, estrogen variations and visceral function. We asserted that the points that we decided to use were specific but other points, like those reported in Habibabadi’s study (13), could also have been tested and should be compared in further studies.

Another study evaluated the stimulation of the concha using electric device and showed that this nonspecific stimulation at 1 Hz for 4 h per day of the vagal afferents of the ear induces an absolute reduction in headache days and results in an improvement in the quality of life as assessed by MIDAS (29). Even if this study used the vagal stimulation component of auriculotherapy, its modality is too different from ours to be compared.

### 4.3. Additions to knowledge of the subject

Our results are contrasted with the absence of a significant difference in the main outcome (number of days with migraines and non-migraine headaches) and several secondary outcomes (number of days with migraine, sum of the pain intensities of all migraines and non-migraine headaches, and total number of analgesics taken, other than triptan, were similar between groups) while we observed a lower number of days with non-migraine headache, less triptan intake, and decreased Midas score when patients received auriculotherapy. This raises the problem of the choice of the primary endpoint.

The discrepancy between the evolution of the number of headache days and triptan consumption can be explained by the observed tendency to less intensity of pain in the treated group. Consequently, we can postulate that although a migraine was present, there was no need to take a specific medication.

Migraine is responsible for a detrimental effect on work-related and everyday life activities (30) and the significant improvement in quality of life in the treated group evaluated using the Midas global score and its subscore evaluating the lost time productivity at work is of major importance. Finally, auriculotherapy allows a significant decrease in non-migraine headaches which possibly plays a role in the improvement of quality of life.

### 4.4. Weaknesses of the study

Our study suffers from some limitations.

First, there is no sham control group to consider the placebo effect on auriculotherapy. Placebo sham auriculotherapy procedures include placing needle in locations that are not specific of the pathology (« hand » or » foot » for example), which could help to determine the efficiency of the chosen points and it could have been interesting to complete our study with a “non-specific points control group.” Placebo sham auriculotherapy also includes non-penetrating “placebo” needles or simple seeds. These techniques could *per se* induce a real effect via the activation of afferent nerves. A “good” placebo should mimic the real treatment. This could be achieved if we had used cryo-auriculotherapy. In this case, an empty cartridge dispensing only the propulsion gas could fake the real treatment. On the other hand, blinding the therapist is difficult to achieve, but perhaps not impossible. A fully trained therapist could, according to a random code, mark the placebo or real points with a felt pen in the patients’ ears, subsequently, a blinded research nurse who is taught how to place the needles but does not know the location of auriculotherapy points, could insert the needles.

We decided to evaluate the patients during this initial part of the treatment, but regarding the chronicity of the migraine, it could have been interesting to evaluate the long-term evolution of the patients. Of importance is the fact that almost all treated patients wanted to pursue auriculotherapy after the end of the study.

Systematic use of an electric detector has been discussed above and should be improved in further studies.

We were surprised by the difference between the characteristics of the patients used to calculate the number of patients and those of the patients included in the study, the latter having much more frequent migraines. This is probably due to a change in patient recruitment, with the more severely affected patients being recruited and the others receiving advice from their general practitioner. In any case, this led us to recalculate the number of patients to be included by considering the characteristics of the patients at the time of their inclusion in the study. By making the same hypotheses, and in particular that of a reduction by half of the number of days with painful episodes of migraines and non-migraine headaches in the treated group, we come to a total of 75 patients (25 in sham group and 50 in auriculotherapy group). These numbers are close to that calculated to build our study.

Finally, regarding the cost of this pathology in terms of loss of workdays and of triptan use, it would have been interesting to complete our methodology with a medico-economic study (31).

### 4.5. Conclusion

Many studies have shown that migraines and non-migraine headache are undertreated, that direct and indirect costs are major, and some have proposed that structured headache services would be cost-effective (32). As auriculotherapy is simple to replicate, free of major side effects, and well accepted by the patients, it could be an important help in structured headache services. However, our study shows contrasted results. Auriculotherapy failed to decrease the number of days with migraine and non-migraine headache but allowed a decrease in triptan intakes and improved the quality of life of patients with migraine. Further studies are necessary to precise the modalities of the treatment.

## Data availability statement

The raw data supporting the conclusions of this article will be made available by the authors, without undue reservation.

## Ethics statement

The studies involving human participants were reviewed and approved by Ethical Committee Kremlin Bicetre, Paris, France. The patients/participants provided their written informed consent to participate in this study.

## Author contributions

MM-C, SM, Md’U, DE, AS, CC-C, and BS contributed to conception and design of the study. MF and MG contributed further insights. NL performed the statistical analysis. MM-C, MF, NL, and MG wrote the first draft of the manuscript. All authors contributed to manuscript revision, read, and approved the submitted version.

## Funding

This work was funded by Hôpital Foch and by Fondation APICIL (grant number 980.17; March 22, 2017).

## Conflict of interest

The authors declare that the research was conducted in the absence of any commercial or financial relationships that could be construed as a potential conflict of interest.

## Publisher’s note

All claims expressed in this article are solely those of the authors and do not necessarily represent those of their affiliated organizations, or those of the publisher, the editors and the reviewers. Any product that may be evaluated in this article, or claim that may be made by its manufacturer, is not guaranteed or endorsed by the publisher.
